# Computer-aided chemistry. Part I: Control of the PAR 273 electrochemical instrument using the IBM 9001 laboratory computer

**DOI:** 10.1155/S146392468600024X

**Published:** 1986

**Authors:** Tebello Nyokong, Martin J. Stillman

**Affiliations:** Department of Chemistry University of Western Ontario London Ontario N6A 5B7 Canada


Journal of Automatic Chemistry/*Journal of Clinical Laboratory Automation*, Vol. 8, No. 3 (July–September 1986), pp. 122–133

### Computer-aided chemistry. Part I: Control of the PAR 273 electrochemical instrument using the IBM 9001 laboratory computer

**Tebello Nyokong and Martin J. Stillman***

*Department of Chemistry, University of Western Ontario, London, Ontario, Canada, N6A 5B7*

### Introduction

The use of high-power, desk-top computers to provide real-time, interactive control of low-cost instruments commonly found in the chemical laboratory will clearly be an area of dramatic development in the near future. Presently, computer programs implementing such computer control fall into one of three main categories by providing either: (1) direct control of the individual operations, with the result being displayed on the instrument for the user (or transferred to a sequential device such as a printer or 'slaved' host computer); (2) 'batch' control, where a series of steps are programmed for a single operation which once initiated runs to completion before user intervention is possible, with the result again being displayed at the end of the experiment as in (1); or (3) a full interactive control of the experiment with real-time display of instrument parameters together with the data that has already been acquired.

One of the most important features of the real-time, interactive control of an instrument with a computer program is the capability of using the program to alter the instrument's operational settings during the course of the experiment. With this type of structure in place, it is relatively straightforward to offer the user a customization option which allows the use of pre-programmed sequences for more complicated methods and special default parameters for individual users. The next stage of development of such a program would be to offer routines that optimize the quality of the data by suggesting modifications be made to the instrument's settings and, in some instances, the chemical method to be used; the program would base its decisions on a data bank of stored methods and past results. Finally, it is possible to develop interactive feedback routines that operate once the experiment has been started, whereby the program indicates to the user possible changes in instrumental settings that will enhance the quality of the data collection. These elaborate features do require high-speed, high-power central processor units, such as the Intel 80286 and Motorola 68000, and the routine availability of random access memory that is greater than 1 megabyte, together with high-resolution cathode ray tube (CRT) displays (>500 by 300 pixels). Because such computers are now relatively inexpensive, interactive control of experimental instruments by desk-top computers has become a reality.

The use of computers in electrochemistry has grown significantly in the past few years. Of all branches of electrochemistry, cyclic voltammetry has received the greatest attention, since a complete analysis of a cyclic voltammetric experiment requires the quantitative measurement of several parameters which affect both the magnitude and shape of the current-potential curve. A computerized system for the acquisition and analysis of the data from linear sweep voltammetry and cyclic voltammetry has been described [1 and 2], while a complete analysis of numerous parameters calculated from the cyclic voltammograms have been carried out by several authors [3 and 4]. In controlled potential coulometry or electrolysis, experiments sometimes require relatively lengthy electrolysis times (up to 1 h), thus real-time computer techniques which can predict the final value of the charge without necessarily requiring that the electrolysis proceeds to completion have been developed [5]. Computers have also been used to synthesize the complex waveforms used in differential pulse voltammetry [6]. However, no description of a completely interactive, real-time computer program for use in the control of electrochemical equipment appears to have been published to date.

Recently-introduced electrochemical instruments have been designed to include extensive capabilities for control by external computers. One such instrument is the Princeton Applied Research (PAR) model 273 electrochemical system. This paper describes the software interface of a powerful microcomputer based on the Motorola 68000 microprocessor, to this sophisticated electrochemical instrument, which is itself completely controlled by a microprocessor. The resultant program, ELECTRA, provides 'hands-on', real-time control of the instrument, using software-programmable 'soft keys' to select action from a range of menus and multiple 'windows' within the two display pages of the CRT RAM. In this way the user can readily alter the parameter settings while the data being acquired is displayed in real time. The electrochemical experiments supported are cyclic voltammetry (CV), differential pulse voltammetry (DPV), controlled potential coulometry (CPC) and controlled potential electrochemistry (CPE). Real-time graphical display of the data from for DPV, CPC and CPE experiments is provided, while for CV the data is displayed immediately after acquisition. The program is written so as to anticipate, prevent and even warn against errors due to the operator requested parameters, thus making it ideal for use in routine electrochemical

* Address correspondence to this author.

analysis. ELECTRA represents the first step in the development of a series of programs that will eventually offer interactive comment on the chemistry that is to take place, in addition to controlling the instrument.

### Implementation of the program
### The computer: the IBM S9001

The main factors which were considered in the choice of a computer for control of the electrochemical system were the requirements of a high-resolution graphics display, fast data transmission rates, large memory capability (>500 K bytes) and the availability of device driver routines to high-level languages. A high-resolution graphics display is essential in electrochemistry as the shapes of the curves can provide considerable information about the properties of the electrode reaction [6]. Fast transmission rates are needed to speed up the experiment, because in many instances the properties of the electrode or the electrode reaction itself can change by an appreciable amount with time. This can be the case during the mesurement of electrochemical parameters of a solution whose constituents adsorb on to the electrodes, thus dramatically altering the electrode surface. Because it is much easier to write and debug complex programs when a high-level language is used, a computer system that offered languages in addition to BASIC was chosen.

The Motorola 68000-based IBM S9001 Laboratory Computer fulfilled all of the above requirements. The computer can be equipped with up to 4 megabytes of memory (RAM), in this case, memory of 1·25 M bytes was used. The standard IEEE 488 parallel port in this computer can be used to attach one or more user devices. The IBM S9001 is equipped with a high-resolution (768 horizontal by 480 vertical pixels) graphics display. It is also equipped with software programmable keys located under the display, which are particularly useful for simplifying operator usage in complex programs where multiple menus are required. The IBM S9001 series computers can run both Pascal and Fortran 77 compilers under CSOS 1·13. These compilers are fully-configured versions of the respective language, however, in addition and significantly, an extensive repertoire of device handlers and system routines are callable directly from the high-level language. The availability of these handler routines greatly reduce the time taken to write programs that are as complicated as ELECTRA. No assembler language routines were needed in ELECTRA.

### The electrochemical equipment: the PAR 273

The PAR 273 Potentiostat/Galvanostat electrochemical system is a new product of EG and G Instruments Division Canada Ltd. The approach of the design of the isntrument is to provide the user with complete control of electrochemical experiments via a host computer. The PAR 273 can be interfaced to a wide range of computers through its IEEE 488 or RS232C ports [8] or to the EG and G PARC model 1000 system processor through a commercial parallel port. Interfacing the PAR 273 through either the IEEE 488 or the RS232C ports allows for complete remote control of the instrument by using

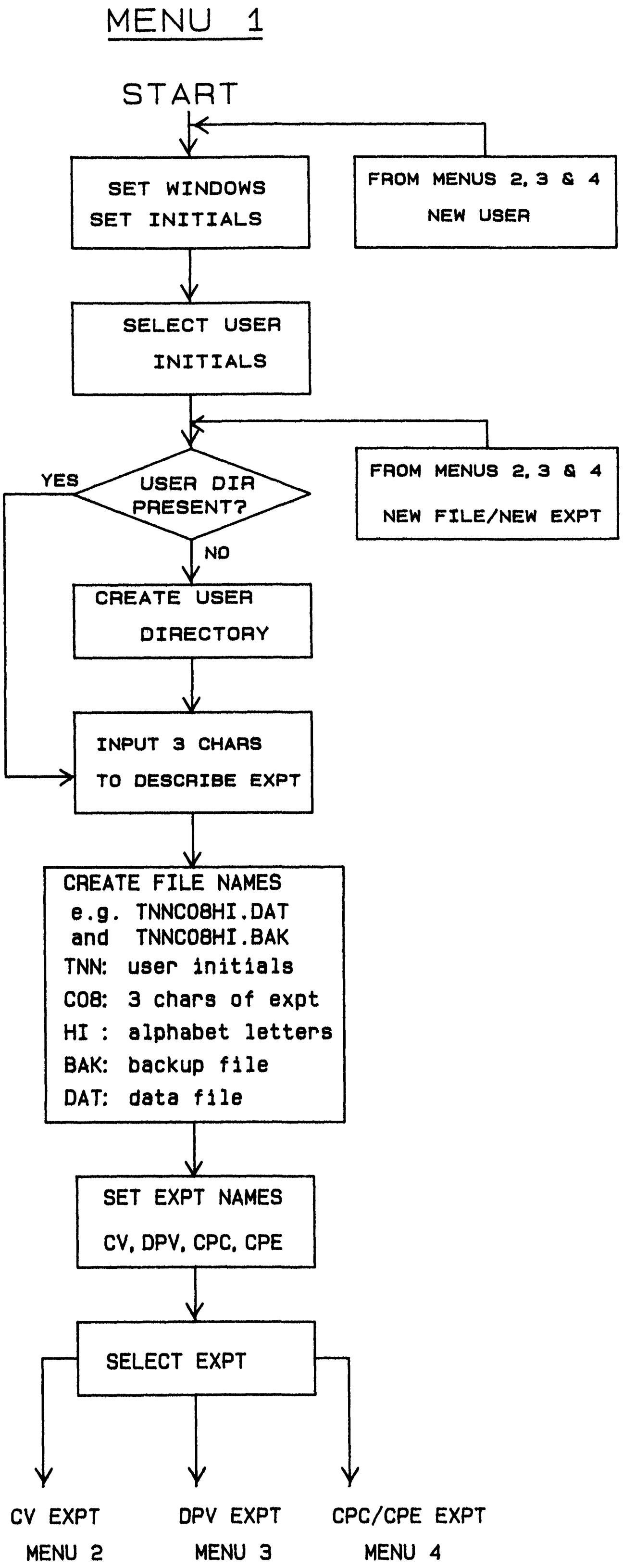

*Figure 1. The flow chart for the program ELECTRA.*

*(a) The PAR initialization and the file structure part; MENU 1.*
*(b) The cyclic voltammetry part; MENU 2.*
*(c) The differential pulse voltammetry part; MENU 3.*
*(d) The controlled potential coulometry and electrochemistry part; MENU 4.*

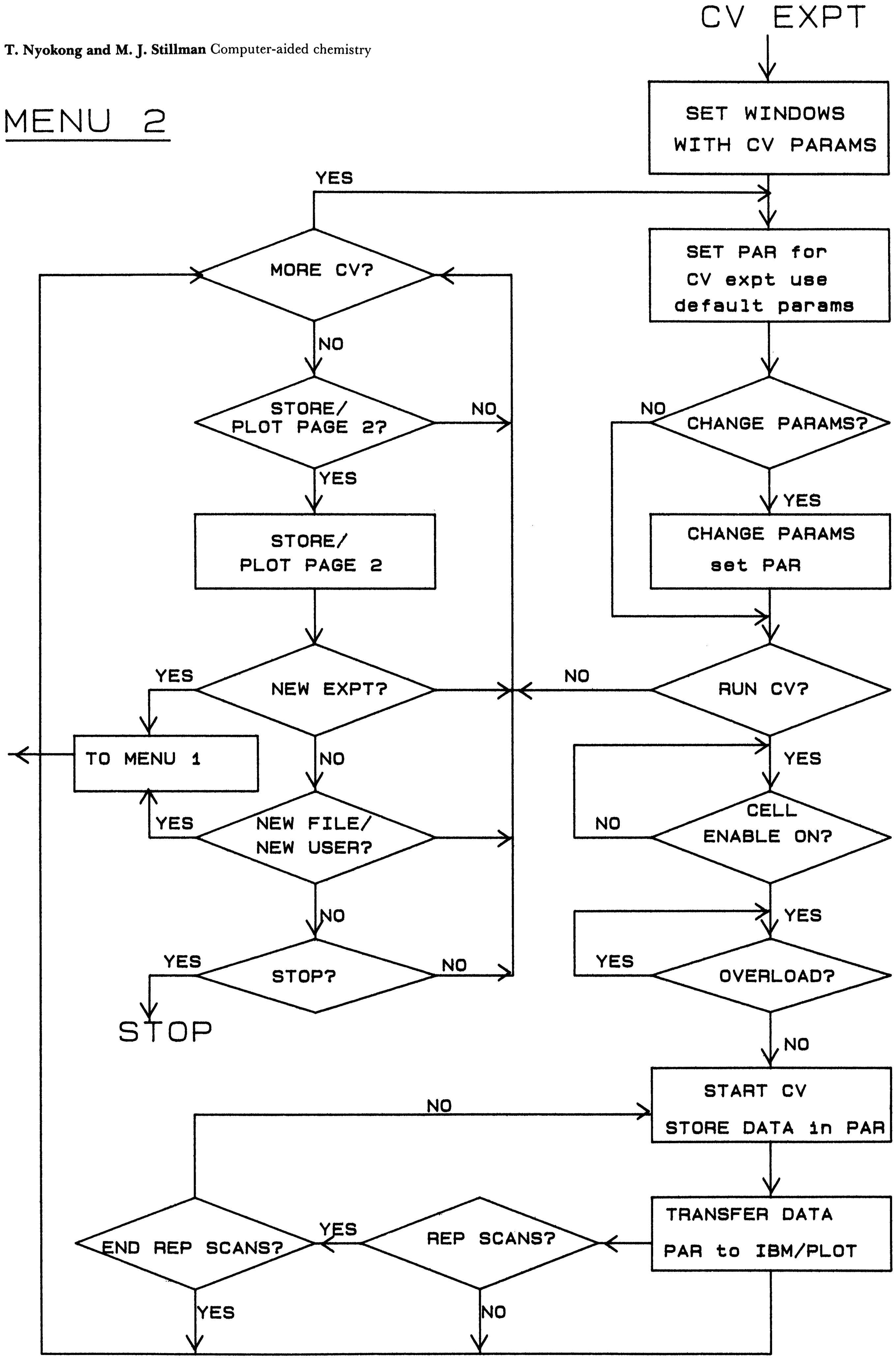

*Figure 1 continued.*

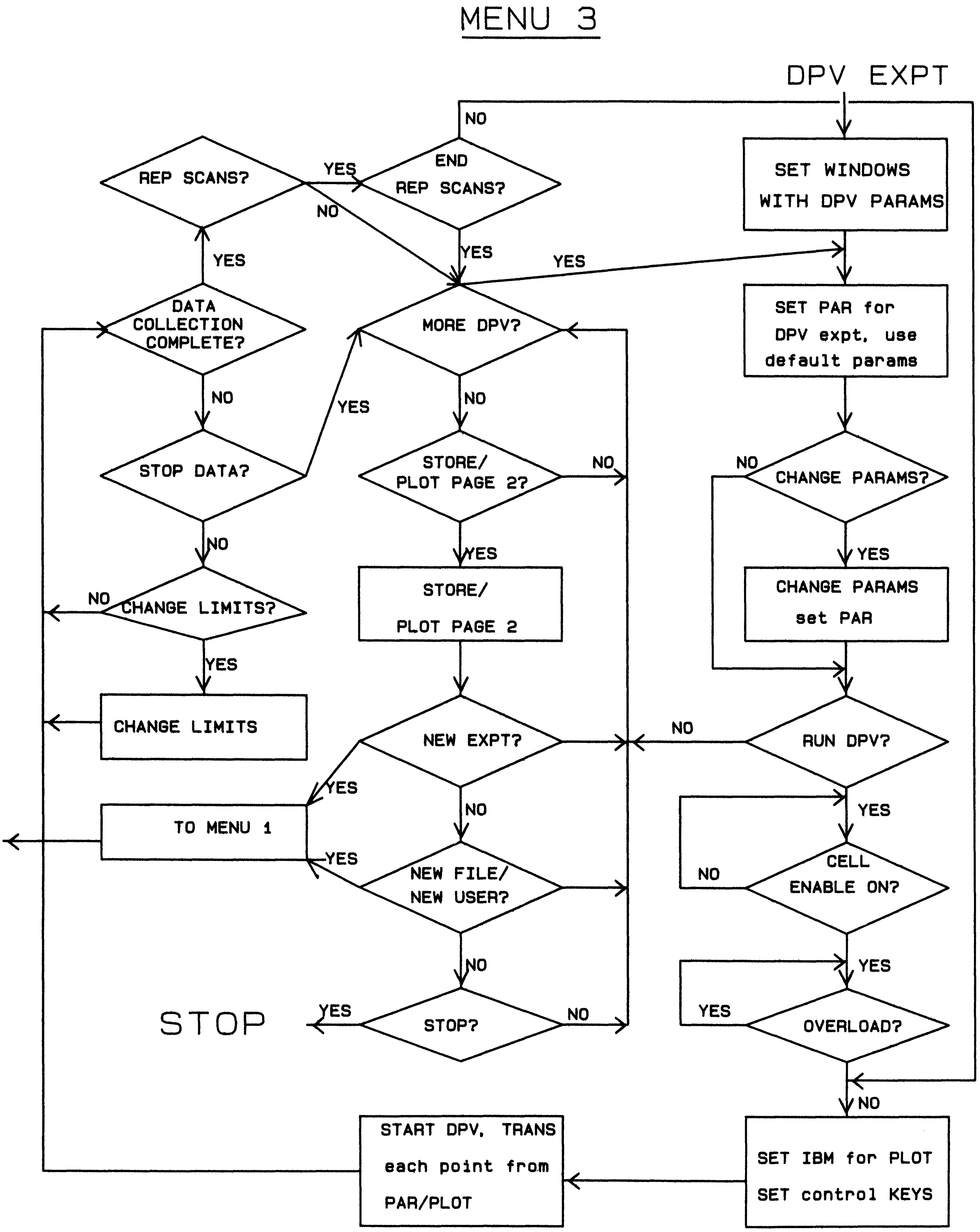

*Figure 1 continued.*

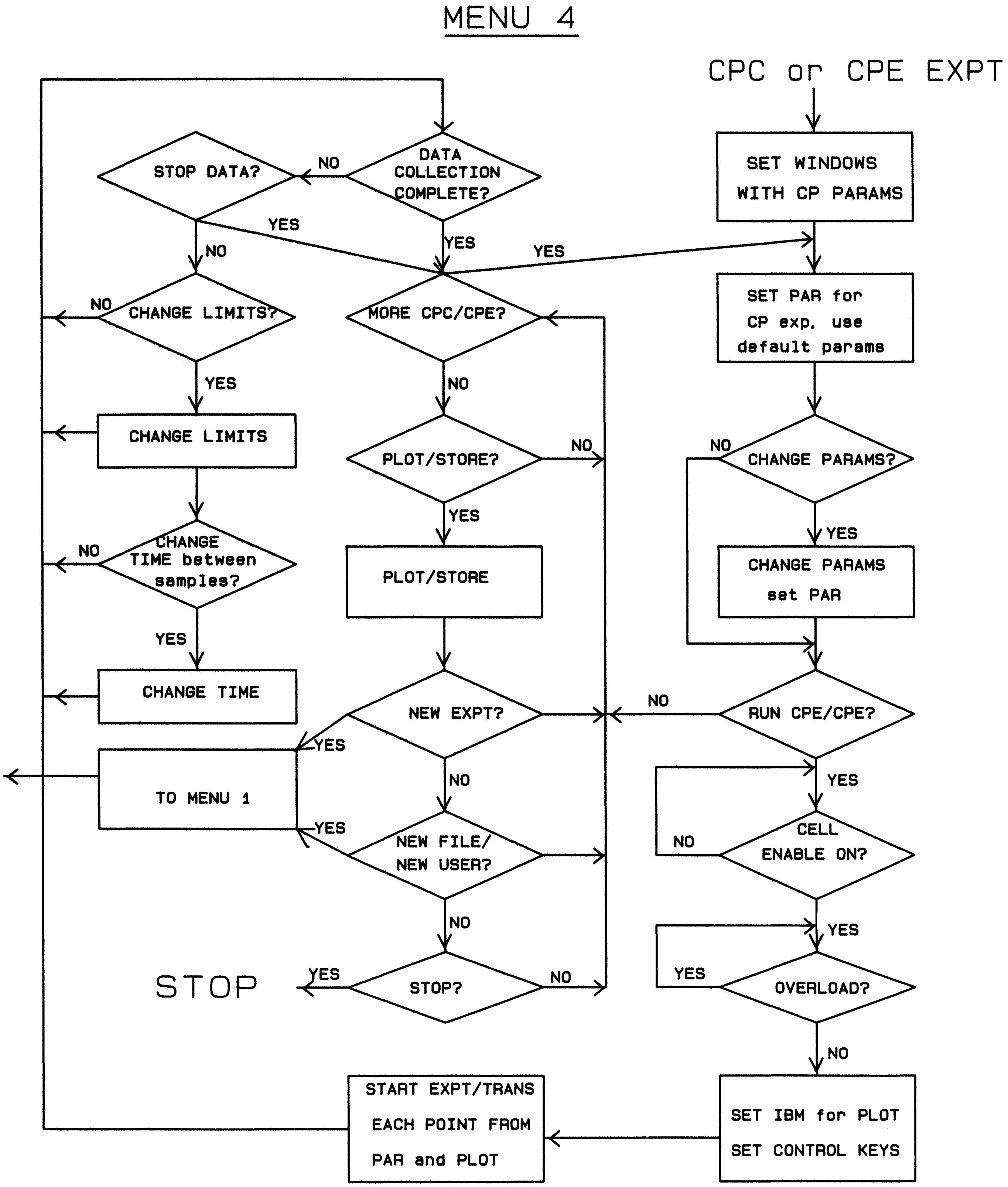

*Figure 1 continued.*

internal mnemonic statements specially developed for PAR 273 electrochemical measurements. These statements provide access to all the front-panel functions, and allow control of internal data storage and manipulation. The PAR 273 is equipped with two 14-bit digital-to-analogue converters for waveform generation, and a 12-bit analogue-to-digital converter to measure current and potential. The on-board microprocessor performs the experiment that is defined by the command set and on-board memory stores the pre-set parameters and data (up to 6144 data points can be stored). Once stored, the data can be transferred to the host computer for plotting or processing. The PAR 273 is also capable of directly reporting the points to the host computer as they are acquired, thus allowing for real-time acquisition and display of individual data points by the host computer.

For the use described in this paper, the PAR 273 was interfaced through the IEEE 488 port. The user selectable IEEE 488 port parameters are the LISTEN and TALK addresses, the test echo mode and the terminator. The host computer communicates with the PAR 273 through the listen and talk addresses. When the test echo mode is on, every character transmitted or received through the IEEE 488 port is echoed to the RS232C port. This is particularly useful during program development, since if a CRT terminal or a printer is connected to the RS232C port, the programmer will see all communications between the host computer and the PAR 273. The terminator on the PAR 273 IEEE 488 port must be selected according to the requirements of the host computer. A carriage return and a carriage return with line feed terminators are provided. The difference between the IEEE 488 and RS232C communications as implemented in PAR 273 is that when the PAR 273 is controlled by the front panel (in the Local mode), the IEEE 488 port is inactive, only becoming active during REMOTE control. The RS232C serial port on the other hand, is always active, thus making it possible to use both the IEEE 488 and RS232C ports simultaneously.

In addition to being controlled from the host computer, the CV, CPC and CPE experiments can also be run directly from the front panel of the PAR 273. However, the DPV waveform can only be generated by remote control from the host computer, since this experiment involves applying a potential pulse for a specified length of time, a feature which cannot be performed by the front-panel operation of the instrument. The PAR 273 features an alphanumeric liquid crystal display which is used to show real-time current and potential values and the experimental settings during the front-panel operation and error messages during remote control [8].

There are three electrodes in typical cells used in electrochemical measurements, the working electrode, at which the reaction of interest occurs, the reference electrode, which provides a known potential, and an auxiliary electrode through which the current is passed to or from the working electrode. If the reference electrode is placed anywhere but exactly at the working electrode surface (which is difficult), there will be some uncompensated solution resistance which will become part of measured potential [6], resulting in an uncompensated potential drop. This is especially critical in non-aqueous solvents which have low dielectric constants and, hence, inherently low solution conductivities. The solution resistance must be taken into account in order to obtain meaningful potential values. The PAR 273 Option 97 IR compensation system carries out this function and this was installed in the PAR 273 electrochemical instrument.

### The software

The program, ELECTRA, is divided into two modules: (1) a data handling module that incorporates the display of menus and data on the CRT, and data storage in disk files; and (2) an instrumentation module, described below in the Discussion, that handles instrument control and data acquisition. In this paper we will discuss features of these modules separately, because the effect of the division within the software is to allow future software to be written rapidly following the same style of on-screen control through soft-key menus and graphical data presentation and the same, common format for the data in the disk files.

### Data handling

Major advances in the complete control of chemical instrumentation by desk-top computers will only be achieved when the implementation of the software control is sufficiently 'user friendly' that no in-depth familiarity is requried with either the programming language or with all the specific details of the software used to run the instrument. Thus, the software should use menu-driven commands via on-screen selection. The best implementation of such a method, but the most complicated to emulate, is the pull-down menu popularized by Apple Computers Inc. on their Macintosh microcomputer, which uses a pointing device such as a mouse or roller ball. Alternative approaches use permanently labelled function keys on the keyboard or software-labelled function keys ('soft-keys'). ELECTRA makes extensive use of 10 software-programmable keys located on the CRT unit, immediately below the screen of the IBM S9001 computer. The menus available are displayed as 12 character labels just above each key. These keys are relabelled throughout the running of the program. Examples of their use include being set as a means of identifying the user, selecting the experiment to be carried out, selecting running parameters and determining the course of the experiment. Because these menus display the only responses possible at any one point in the program, the user does not have to remember specific responses or, even worse, specific, cryptic mnemonics; thus keyboard errors are reduced and response time is greatly improved.

It is likely that, in the future, programs running on computer-controlled instruments will offer the user a very sophisticated array of operational techniques, amongst which will be some form of automatic assessment of the chemistry taking place using past results that are stored on disk. Considerably more complicated graphical output designs will be needed compared with simple scrolling messages, to cope with the rapid and detailed information transfer required between user and computer. In general, then, the software should allow the user to set up and run the instrument without recourse to a manual after the initial familiarization session, and, later, as a means of checking that full use of both computer and instrument is being made.

An important design decision, but one that is frequently overlooked, is the future accessibility of the stored data on disk. With the advent of optical storge disks and local area networks, scientific users will probably want in the future to leave their data on-line. This will create considerable difficulties where large numbers of files are in use at any one time, as is the case when a data acquisition program sets up new files for every experiment or even every day. The program ELECTRA offers a novel solution to this problem by establishing user-tagged system directories that log the file names of all previous data files, together with date, time and volume name of the data file. The program then constructs new file names by combining the initials of the user, a user-entered, three-letter descriptor and an alphabetic running sequence. A system character incorporated into the file name allows several different programs operating on different computers to generate unique file names. Our data processing programs then use *directories* of file names to guide the entry of data sets. Thus the user can construct directories in an editor and make the directory name a mnemonic for the data held within the files.

The use of multiple pages for graphical information also provides a powerful technique for assembling considerable amounts of data together with instrumental control parameters. In ELECTRA, one page is used to display all instrument settings and a reduced size plot of the data, while a second graphics page is used to display the data using the full screen, shuttling between the two pages is extremely fast and is achieved by pressing the PAGE soft-key.

The flow chart for the ELECTRA program is shown in figure 1. There are four parts to it: (*a*) represents, mainly, the data handling module, and parts (*b*)–(*d*) represent the instrument control modules. The initialization of the computer, identification of the user and the construction of the file name is shown in figure 1(*a*), while (*b*), (*c*) and (*d*) show details of the program for initialization and operation of the PAR 273 for cyclic voltammetry (CV), differential pulse voltammetry (DPV) and controlled-potential coulometry (CPC) and electrochemistry (CPE), respectively.

A total of 20 experiments, comprising up to 1000 points each, can be recorded and stored before a new file is needed. There is a choice of presetting the total number of scans desired, with the same parameters, and scanning will proceed without operator intervention. The program also allows the user to record the base-line (usually solvent plus electrolyte) and subsequently subtract it from the data. At any time, the user can plot some or all of the scans on the second page of the CRT display. This gives a better view since a full screen graphics winow is used. The plots can readily be printed on the IBM S9001 integral printer, if wished, using the screen print capabilities of the computer. All, or only a selected number of scans can be stored on disk. In this way only the data which the user thinks are useful need to be stored. The data are stored with all the parameter information that is necessary for the description of the experiment, and it is stored in the form that is ready to be plotted with the Hewlett-Packard 7550A graphics plotter, using the program Spectra Manager (W. R. Browett and M. J. Stillman, unpublished). There is a choice at any time during the operation of the program, to change the type of experiment, to alter the file names, to discard or store the data, or to view the data on the second page of the CRT display.

### Instrument control and data acquisition

*Initialization*

At the start of an experiment the program sets up the graphics display windows and labels the software defined keys with a variety of user initials (see figure 1[*a*]). Selection of one of these sets of initials triggers the file name calculation followed by the establishment of two files, a main file (⋆.DAT) and concurrent backup file (⋆.BAK). Soft-keys which permit the user to select an experiment are then labelled and the parameters, together with their default values, are displayed on the screen. The PAR 273 is reinitialized at the start of each experiment in order to restore the default values of the parameters. The IR compensation mode is also set at the beginning of each experiment. Before curve acquisition can commence the program checks to see if the CELL ENABLE switch, which connects the PAR 273 to the external cell, on the front panel of PAR 273 has been turned on and, if not, the user is asked to turn it on; turning on the CELL ENABLE is the only operation on the PAR 273 that cannot be done by the computer. The program also flags current and potential overload conditions which require user intervention using the appropriate soft keys. All the messages to the user are displayed in inverse video for ease of recognition.

The program described here has been used routinely to study the electrochemistry of porphyrins and phthalocyanines. Most of these complexes are insoluble in water and hence the use of the universally-accepted, water-based external electrodes such as the normal hydrogen electrode (NHE) or the saturated calomel electrode (SCE) would include unknown amounts of the liquid-junction potentials. This would result in electrochemical parameters which could not be related to each other and would be difficult to reproduce. In order to avoid this problem, the ferricinium/ferrocene couple was used as an internal standard [7] and silver wire as an internal reference electrode. Thus the actual half-wave potentials shown on the screen prints (figures 2–4) may vary from run to run since the concentration of silver ions around the reference silver wire may vary; however, the difference beween the half-wave potentials of the complex of interest and the half-wave potential of ferrocene is constant.

*Cyclic voltammetry (CV)*

Cyclic voltammetry involves the measurement of the current ($i$) between two electrodes in a cell as the potential ($E$) is varied from to initial value to a potential past the peak potential and then reversed, normally, back to the original value. For reversible chemical systems, a peak in the current flow will be measured as the potential is varied in the forward direction and another peak of the same magnitude but different sign in the current, will be detected as the potential is reversed. In non-reversible systems no peak is measured during the potential reversal, while in quasi-reversible systems, the magnitudes of the current peaks on forward and reverse directions are different. Cyclic voltammograms are plots of the current versus potential. The most important parameters on these $i$–$E$ curves are (1) the half-wave potential ($E_{\frac{1}{2}}$), measured as half of the sum of the forward and reverse peak potentials ($\frac{1}{2}(E_f + E_r)$) for reversible systems, while for non-reversible systems the measured parameter is the peak potential; (2) the ratio of the peak currents, which should be equal to one for reversible systems and differ from one in quasi-reversible sytems; and (3) the separation of the peak potentials which should be equal to $58/n$ mV for reversible systems, where $n$ is the number of moles of electrons involved in the electron transfer step.

The PAR 273 performs cyclic staircase voltammetry rather than true cyclic voltammetry. The parameters essential for decribing the CV experiment in the PAR 273 system are the initial potential ($E_i$), vertex potential ($E_v$), final potential ($E_f$), the current range, the scan rate and the resolution in points per volt. In normal applications, the initial and final potentials are identical. When the essential parameters have been set by the user, a ramp program is calculated based on the values of $E_i$, $E_v$, $E_f$ and the total number of millivolts spanned. The ramp program consists of only three steps that define the starting point, the first slope and the second slope, thus creating a triangular ramp. Once stored, the ramp can be used repeatedly until the user changes the parameters describing the ramp, at which point a new ramp will be created. In the PAR 273, the initial and final potentials must be within 2000 mV of each other, while the scan rate cannot exceed 8000 mV/s. All the parameters can easily be changed by pressing the appropriate soft keys. Once an acceptable ramp has been created, curve acquisition proceeds on the PAR 273, and when complete the data are transferred to the computer for storing and plotting. The half-wave potential ($E_{\frac{1}{2}}$) calculated as ($E_{\text{oxid}} + E_{\text{red}}$)/2 and the peak width ($E_{\text{oxid}} - E_{\text{red}}$) are displayed on the screen in inverse video for each CV curve (figure 2a). Curve acquisition takes only a couple of seconds; for example, the CV scan from 0 to 2 V and back to 0 V, at a scan rate of 500 mV/s, will take about 8 s, while transferring date through the IEEE 488 port is a much slower process. It is faster to transfer all of the data at the completion of the curve acquisition, rather than transferring it point by point as it is acquired. Once the data is stored in the computer, the user can continue to acquire more curves with the same or different parameters.

Traditionally, when setting up cyclic voltammetry, the user adjusts the front-panel switches while the potential is being cycled and the voltammogram is being drawn on an X/Y recorder. ELECTRA offers a similar method by displaying repetitive scans on the screen, the user can interrupt the scanning sequence to change parameters at any time by using soft-keys. Because chemical reactions sometimes occur following oxidation or reduction it is essential to be able to vary the rate at which the potential is cycled, i.e. the V/s value. The computer program, therefore, must provide the user with the capability of adjusting the ramp rate for $E$ while observing the resultant $I$ versus $E$ plot on the screen. Repetitive traces are laid down so that systematic changes in the curve can be observed. Once the chemical behaviour of the sample is known, then a curve is taken that is stored. ELECTRA implements these criteria as shown in figure 2, which shows a copy of the screen taken during this operation. At this stage in the experiment the user does not need to touch the cell or the PAR 273. In order to achieve the maximum ramp rate of the PAR 273 (8 V/s), the entire cycle is carried out by the PAR's microprocessor, then the data is transferred to the IBM 9001 as a complete curve.

*Differential pulse votlammetry (DPV)*

The differential pulse voltammetry experiment involves increasing the potential steadily in small increments and applying a pulse of fixed height and width at each potential step. The current is sampled immediately before and after the application of this pulse. The DPV experiment was originally designed for mercury electrodes, where the first current sample was taken just before the pulse application on the mercury drop and the second sample late in the pulse just before the drop was dislodged, resulting in a pulse width of 5 to 100 ms. During the application of a pulse on solid electrodes, the current decays to a residual level after about 2·5 ms, hence a pulse width of 2·5 ms is adequate. Differential pulse voltammograms are plots of the arithmetic difference between the two current samples versus the potential. The measured parameters are the $E_{\frac{1}{2}}$ potential (measured as the peak potential) and the width of the peak at half-height, $W_{\frac{1}{2}}$, which is theoretically equal to 90·4, 45·2 and 30·1 mV for $n = 1$, 2 and 3, respectively [6]. The DPV experiment has an advantage over the CV experiment in that large signals can be obtained in the former for very dilute solutions, thus facilitating accurate measurements of the $E_{\frac{1}{2}}$ values. The increase in the relative sensitivity of the DPV experiment is due to the reduced charging current contribution in the measurement.

The DPV experiment is much slower than the CV experiment because there is a pause after the application of the step in potential, although the time at the top of the step is much shorter for solid electrodes than with hanging mercury drop electrodes. Because of this, the computer can obtain the current data on a point by point basis and provide the user with real-time display of the data. Additionally, because the computer has the values of both potentials, the plot of $\Delta i$ versus $E$ used the correct value of $E$, when plotted on an X/Y recorder the $\Delta i$ value is significantly displaced from the actual value of the potential that was used.

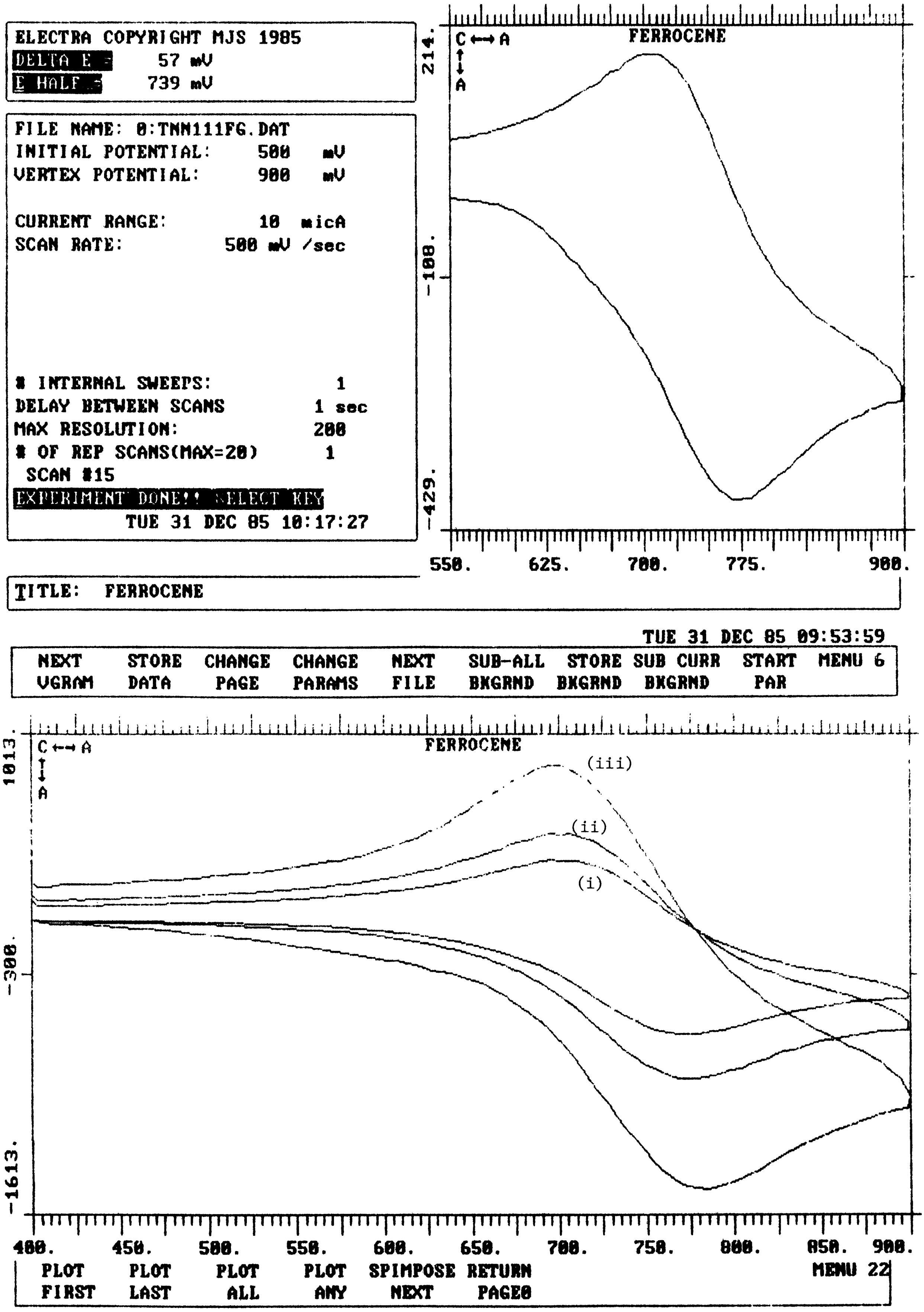

*Figure 2. Screen prints for the cyclic voltammetry experiment for ferrocene dissolved in dimethyl acetamide, using silver wire as the reference and 0·10 M tetrapropylammonium perchlorate as the electrolyte. Units on the plots are, for the Y axis: current ($\times 10^{-2}$) μA and for the X axis: voltage in mV.*

*(a) The first CRT page at the completion of one experiment.*

*(b) The second CRT page showing the effect of scan rate on the shapes of the CV curves:*

*(i) 500 mV/s; (ii) 1 V/s; (iii) 3 V/s.*

*Note that arrows linking the symbols C and A drawn on each plot indicate the directions of cathodic and anodic potential and current flow.*

The parameters necessary to describe the DPV experiment in the PAR 273 system are the initial potential ($E_i$), the final potential ($E_f$), the current range, the pulse height, the time between current samples, the potential increments, and the ramp step size. A ramp is generated by incrementing the initial potential regularly until the final potential is reached. At each potential 100 current samples are taken and a pulse of height normally ranging from 10 to 100 mV, is applied during the last five samples [6] (see figure 3). The reported current is the difference between the current sampled at the last point and the current sampled just before the application of the pulse. In order to speed up the data acquisition process, while the current is being sampled for one potential, the program starts to set up the parameters for the following potential, so that in essence two points are being handled at the same time. As in the CV experiment, each parameter can readily be altered using appropriate soft-keys. Increasing the ramp step size results in fewer data points, hence faster acquisition but with some loss in resolution. Increasing the pulse height to over 100 mV can also result in the degradation of the peak resolution [6]. The span between the initial and final potentials cannot exceed 2000 mV in the PAR 273. Once the ramp has been created it can be used until a new ramp is specified by the user.

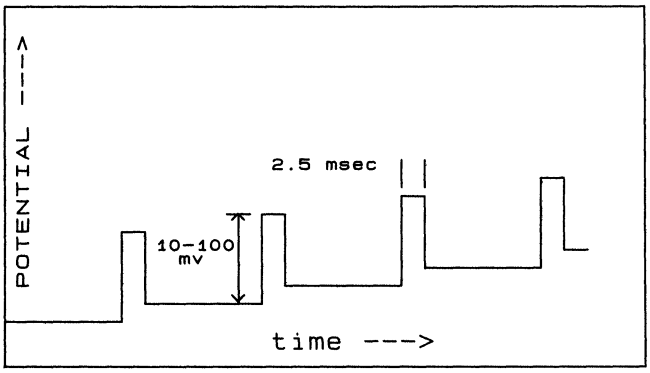

*Figure 3. A Schematic representation of the differential pulse voltammetry potential pulse sequence.*

Each point is transferred directly to the computer as acquired. The data is displayed and plotted in real-time. Figure 4 shows a sample plot from the DPV curve of ferrocene dissolved in 0·10 M tetrapropylammonium perchlorate in dimethyl acetamide. The half-wave

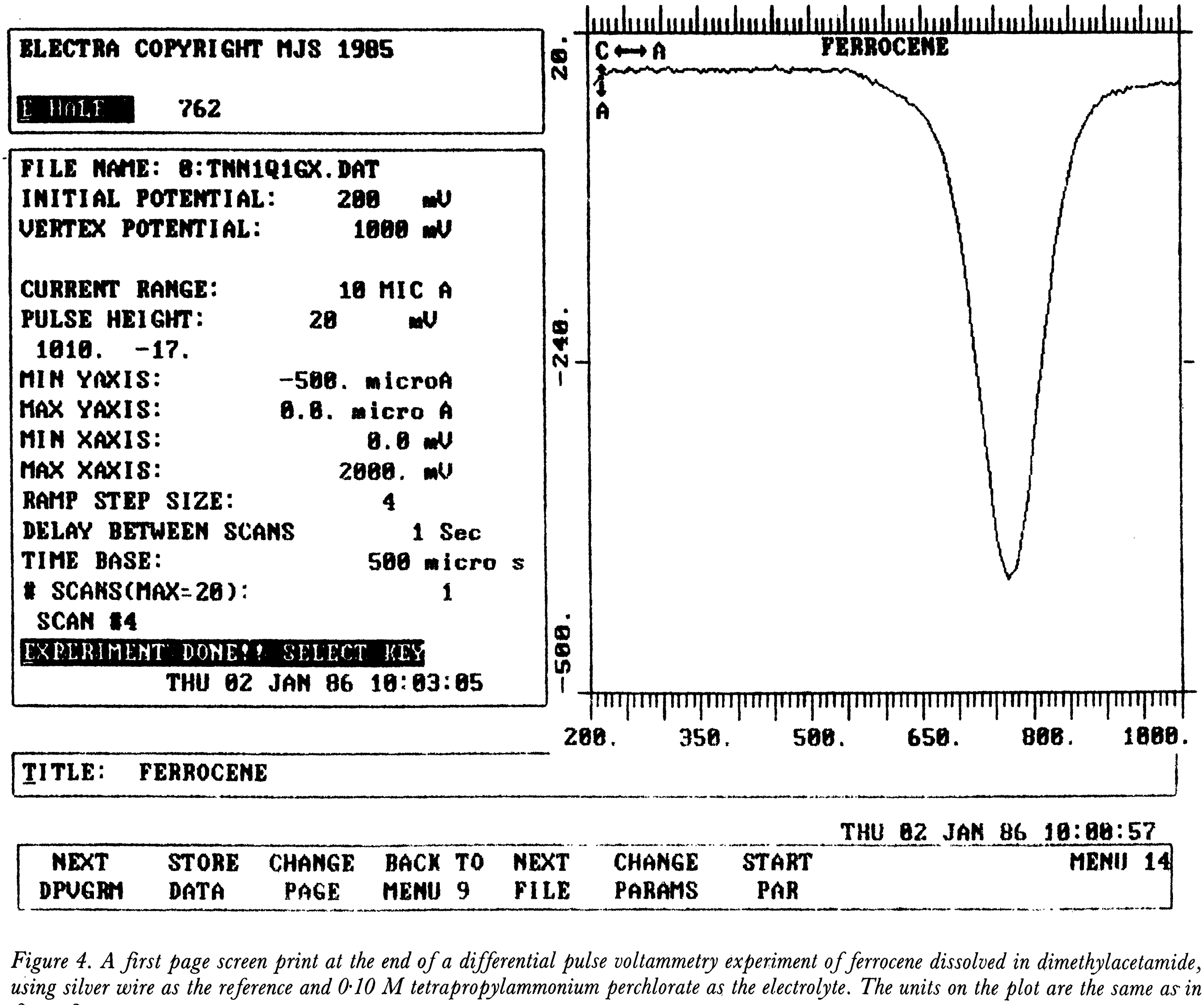

*Figure 4. A first page screen print at the end of a differential pulse voltammetry experiment of ferrocene dissolved in dimethylacetamide, using silver wire as the reference and 0·10 M tetrapropylammonium perchlorate as the electrolyte. The units on the plot are the same as in figure 2.*

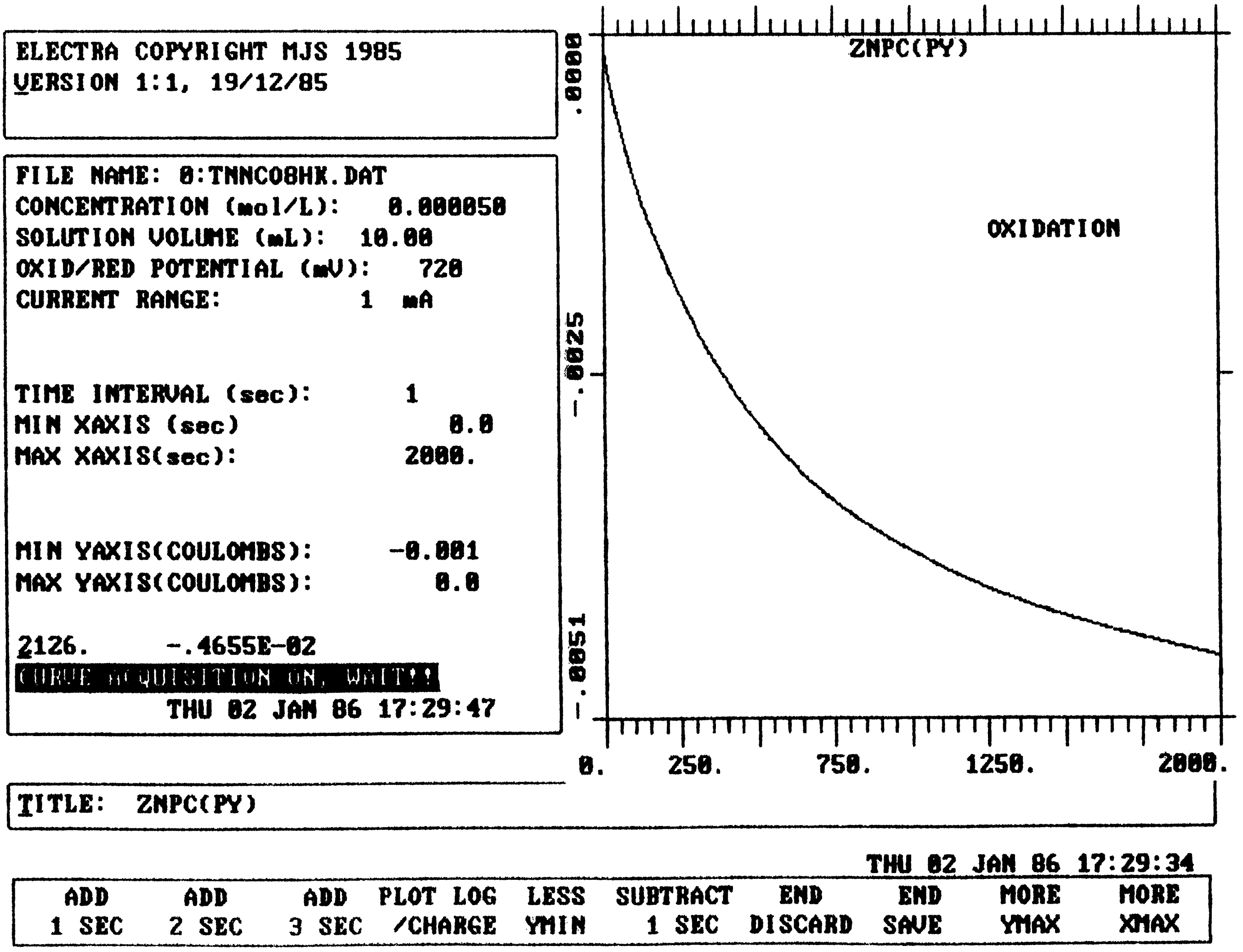

*Figure 5. A screen print taken during the controlled potential coulometry experiment of zinc phthalocyanine dissolved in dichloromethane (at 0·72 V versus silver wire), using 0·05 M tetraethylammonium perchlorate as the electrolyte.*

potential, as $E_{min}$, is calculated and displayed on the screen in inverse video. As the curve acquisition proceeds the user has an option to change the plotting limits for better viewing or to end data collection and to store or discard the data.

*Controlled potential coulometry (CPC) and electrochemistry (CPE)*

The CPC and CPE experiments involve the application of a fixed potential to the cell and the measurement of the variation of current with time [6]. The CPE curve is a plot of this current versus time, while in the CPC experiment it is the integral of the current (charge, $Q$) that is plotted, versus time. Both the current and the concentration decay exponentially with time during the electrolysis, the current eventually attains a residual level. The area, $A$, of the working electrode and the volume, $V$, of the solution are essential in determining the rate of the electron transfer in the CPC and CPE experiments. Electrolysis cells designed to give large $A/V$ ratios result in shorter electrolysis times than those with a lower ratio. Short electrolysis times are useful in cases where the desired electrolysis product can undergo further reactions, since the preparation of high yields of the product is normally the main aim of the CPC and CPE experiments. The shapes of the current–time curves give information about the course of the reaction as the electrolysis proceeds, very complex current–time curves can be obtained in cases where the electrolysis product undergoes further reactions at an appreciable rate during the early stages of the electrolysis. Information about the number of moles of electrons involved in each electron transfer step is provided by the CPC experiment.

These experiments are the simplest to control by computer and the availability of digital data allows for both calculation of the complexity of the reaction and the number of electrons transferred. Because the reactions are slow, and it is the shape of the $i$ versus time or $Q$ versus time curves that provides much of the information to the user, automatic rescaling of the plotted data so that the screen is always filled is most important and represents a significant advantage over traditional displays using X/Y plotters where the Y axis scale is fixed. On-line calculations that fit the measured data to equations that represent the theoretical curve allow for real time predictions of the time course of the electrochemical reaction by superimposing the prediction on the data.

Deviations from this theoretical line give an indication that secondary reactions have begun to be significant.

The parameters essential for describing the CPC and CPE experiments in the PAR 273 system are the oxidation potential, the current range and the time between data samples. In these experiments, a fixed potential is applied to the cell at a preset current range and either the charge or the current is sampled, at a selected time interval, by the computer. The data is displayed and plotted on the screen as it is acquired. Figure 5 shows the CPC curve for zinc phthalocyanine in 0·05 M tetraethylammonium perchlorate in dichloromethane. During the early stages of the electrolysis the charge increases rapidly with time, but later in the electrolysis, as the concentration of the original species decreases, a much slower increase of charge with time is observed, until finally the curve reaches a plateau, when all of the unoxidized species has been used up.

The program allows both the time interval between the acquisition of data points and the range in the X and Y axes of the plot to be altered at any time during the curve acquisition using appropriate soft-keys. Curve acquisition can be terminated at any time and the data kept or discarded as wished. Once a CPC or CPE experiment has been performed, the properties of the solution change and although the program still has the capabilities to run a total of 20 scans, this would involve different solutions, unlike the case for the CV or DPV experiments where the same solution can be used several times. The CPC data is stored in the form ready to be fitted to the least square program, BARD [9]. The results obtained from this program give information about the rate of electron transfer and the number of moles of electrons transferred.

### Discussion

The program ELECTRA described in this paper is made up of two components: (1) a generic data handling unit that provides disk file management and on screen graphical displays; and (2) a driving unit that controls the PAR 273 electrochemical instrument. The first unit provides unique features that greatly improve the ease of use of computer-controlled equipment. Through the incorporation of this unit within the ELECTRA program techniques have been developed that will be required once high-capacity disk drives and laboratory networks are common enough that scientists will want to leave all relevant data on-line for further data processing and plotting. The program exploits the power and graphical display capabilities of the IBM S9001 computer to provide complete, high-speed control of the PAR 273 instrument using the IEEE for data transfer.

The PAR 273 is a state-of-the-art instrument in that it is equipped with one of the latest microcomputer designs, thus allowing for high performance and ease of use. Most low-cost instruments found in the chemical laboratory today are equipped with less-efficient microcomputers, if any. However, even with such an up-to-date instrument there are still significant limitations to the extent of control that can be applied by an external host computer. Clearly, for optimum control capabilities, the internal computer must be able to stack commands from the host and allow use of the host's real-time computing capability during the course of the experiment. This type of interaction requires more extensive use of interrupts and data buffers on the instrument's internal computer in order to reduce the overhead of the communication with the host.

### Acknowledgements

We wish to acknowledge financial support from NSERC of Canada through Operating, Strategic and Equipment grants (to MJS) and the Academic Development Fund at the UWO for an equipment grant (to MJS). We are also grateful to the financial support (to TN) of the Canadian International Development Agency (CIDA) in conjunction with the National University of Lesotho. We wish to acknowledge extensive discussions concerning programming details with Dr William Browett and Mr Robert Kitchenham. The authors are associated with the Centre for Chemical Physics at the UWO.

### References

1. Perone, S. P., Frazer, J. W. and Kray, A., *Analytical Chemistry* (1971), 1485.
2. Dorin, R., *Computer & Chemistry*, **8** (1984), 133.
3. Creason, S. C., Loyd, R. J. and Smith, D. E., *Analytical Chemistry*. **44** (1972), 1159.
4. Witson, P. E., VandenBorn, H. W. and Evans, D. H., *Analytical Chemistry*, **45** (1973), 1298.
5. Stephens, F. B., Jakob, F., Rigdon, L. P. and Harran, J. E., *Analytical Chemistry*, **42** (1970), 764.
6. Bard, A. L. and Faulkner, L. R., *Electrochemical Methods, Fundamentals and Applications* (John Wiley and Sons, 1980).
7. Nyokong, T., Gasyna, Z. and Stillman, M. J., submitted to *Inorganic Chemistry*.
8. EG and G PAR Operating Manual (1985).
9. Kuester, L. J. and Mize, J. H., *Optimization Techniques with Fortran* (McGraw Hill Book Company, 1973), p. 218.

